# 小细胞肺癌的免疫治疗临床进展

**DOI:** 10.3779/j.issn.1009-3419.2022.102.15

**Published:** 2022-06-20

**Authors:** 维威 王, 家齐 张, 单青 李

**Affiliations:** 100730 北京，中国医学科学院北京协和医学院，北京协和医院胸外科 Department of Thoracic Surgery, Peking Union Medical College Hospital, Chinese Academy of Medical Sciences and Peking Union Medical College, Beijing 100730, China

**Keywords:** 小细胞肺癌, 免疫治疗, 临床试验, Small cell lung cancer, Immunotherapy, Clinical trials

## Abstract

小细胞肺癌是一种侵袭性强、预后差的恶性肿瘤，多学科联合的综合治疗是其经典的治疗模式。长期以来，小细胞肺癌的治疗方案一直停滞不前；近些年，免疫治疗药物的初始经验使得小细胞肺癌的治疗出现了新的契机。部分免疫检查点抑制剂的临床试验已经证实了其在小细胞肺癌方面的有效性及安全性；基于Ⅲ期临床试验（Impower133和CASPIAN研究）的结果，目前美国国家食品药品监督管理局已经分别批准Atezolizumab、Durvalumab联合化疗用于广泛期小细胞肺癌的一线治疗。包含免疫检查点抑制剂的不同治疗方案在小细胞肺癌人群的临床试验正积极广泛开展，为小细胞肺癌的治疗提供了不同的视野。本文综述目前小细胞肺癌免疫治疗的临床进展。

## Abstract (en)

Small cell lung cancer is a kind of malignant tumor with strong invasiveness and poor prognosis, and the classic therapeutic modality of the disease remains multidisciplinary and comprehensive treatment. Treatment options for small cell lung cancer have been stalled for a long time, and new opportunities have emerged in recent years due to the development and initial experience of immunotherapeutic drugs. Clinical trials of some selected immune checkpoint inhibitors have confirmed the efficacy and safety in small cell lung cancer. Based on the results of phase Ⅲ clinical trials (Impower133 and CASPIAN), Atezolizumab or Durvalumab in combination with chemotherapy has been approved by the U.S. Food and Drug Administration for the first-line treatment of extensive-stage small cell lung cancer. Clinical trials involving immune checkpoint inhibitors are being actively carried out and provide different perspectives for the management of small cell lung cancer. This article aimed to review the clinical progress in immunotherapy of small cell lung cancer.

小细胞肺癌（small cell lung cancer, SCLC），是一类低分化恶性上皮性肿瘤，属于高级别神经内分泌癌，目前占所有肺癌的15%左右；其发病率可以反映吸烟的流行情况，这种相关性一般滞后30年^[1]^。美国监测、流行病学和结果数据库资料^[2]^分析显示，2016年美国SCLC的发病率为5.48/100, 000人，其发病率及死亡率较前均有所下降。国际肺癌研究协会的资料^[3]^显示，Ⅰ期、Ⅱ期、Ⅲ期和Ⅳ期的SCLC患者2年总体生存率分别约为73%、54%、23%和8%。西班牙的数据^[4]^指出，约20%的SCLC患者在确诊时存在脑转移，96.7%的SCLC患者可以观察到病变转移；其中位总生存期（overall survival, OS）约9.5个月，1年和2年生存率分别为38.9%、14.8%。SCLC恶性程度高、侵袭性强，容易出现转移，预后差。近些年，由于包含免疫治疗的综合治疗模式的发展，SCLC患者的生存预后得到一定程度的改善。本文综述目前SCLC免疫治疗临床进展。

## 1 SCLC的病理分类历史

SCLC，曾被称作燕麦细胞癌，最早由Barnard^[5]^于1926年提出，其神经内分泌特性于20世纪60年代前后被发现^[6]^，而且与鳞状细胞癌具有一定的同源性。1967年第一版世界卫生组织（World Health Organization, WHO）肺部肿瘤组织分类中，肺癌被大致分为SCLC和非小细胞肺癌（non-small cell lung cancer, NSCLC）；1999年发布的第3版WHO肺及胸膜肿瘤组织分型^[7]^提出与吸烟相关、可能可逆的神经内分泌增生小体可能是SCLC的前驱病变；SCLC属于神经内分泌肿瘤的一种亚型；目前，WHO组织分型明确将SCLC作为神经内分泌肿瘤的亚型进行分类。SCLC的病理诊断一直延续20世纪90年代的组织细胞形态学诊断标准^[8, 9]^，手术切除标本是标准的诊断组织来源；其组织上表现为原发性肺上皮恶性肿瘤，肿瘤细胞胞浆稀少、细胞边界不清、细颗粒状核染色质且无核仁或者核仁不明显，细胞体积小于3个静息淋巴细胞^[10]^。对于小标本而言，可以结合免疫组化（高表达Ki-67以及神经内分泌分化标记物如突触素、嗜铬蛋白A、神经细胞黏附分子1和胰岛素瘤相关蛋白1）辅助诊断，相比于其他神经内分泌肿瘤，SCLC具有较高的核分裂，常见坏死。根据是否合并存在其他成分，SCLC分为单纯性SCLC及混合型SCLC。混合型SCLC是指SCLC同时合并任何一种NSCLC成分，包括鳞癌、腺癌及大细胞神经内分泌癌甚至少数其他类型；除了混合大细胞神经内分泌癌要求其占10%以上外，其他混合型对于其他成分的比例无具体要求。2021年新版的WHO肺部肿瘤组织分型新增了SCLC分子分型的介绍，这些分型可能影响治疗方案的选择^[9]^。

## 2 SCLC的免疫治疗

SCLC的免疫治疗可追溯到20世纪70年代；1975年Hornback及其同事^[11]^在第17届美国放射治疗协会的年会上报道了29例燕麦细胞肺癌患者的综合治疗结局，其中提及卡介苗免疫治疗，这可能是SCLC免疫治疗的雏形。近年来，免疫检查点抑制剂（immune checkpoint inhibitors, ICIs）的发现为肿瘤免疫治疗带来了革新的局面。SCLC的现代免疫治疗经验来源于NSCLC。目前主要的免疫检查点包括细胞毒性T淋巴细胞相关抗原4（cytotoxic T lymphocyte-associated antigen 4, CTLA-4）、程序性死亡蛋白1（programmed cell death protein 1, PD-1）以及程序性死亡受体1（programmed cell death ligand 1, PD-L1）；针对不同的免疫检查点的ICIs能阻断相应的信号传导通路，重新激活T细胞的生物活性，抑制肿瘤细胞的增殖。目前临床上常见的ICIs包括PD-1抗体（Pembrolizumab、Nivolumab等），PD-L1抗体（Atezolizumab、Durvalumab等）及CTLA-4抗体（Ipilimumab、Tremelimumab等）。

临床上，SCLC的治疗仍基于其分期而论；目前SCLC主要分为局限期SCLC（limited stage SCLC, LS-SCLC）和广泛期SCLC（extensive stage SCLC, ES-SCLC）。

### 2.1 LS-SCLC的免疫治疗

由于放化疗联合预防性颅脑照射的标准治疗模式对LS-SCLC能起到相对较好的疾病控制，因此，免疫治疗在标准治疗后的LS-SCLC患者中尝试不多。LS-SCLC免疫治疗的临床试验较早见于1985年报道^[12]^，患者接受放化疗后随机分配进行联合或不联合卡介苗免疫治疗，其结果表明，联合卡介苗的免疫治疗并不能延长患者生存。2020年，Welsh等^[13]^报道了一项单中心的Ⅰ期/Ⅱ期临床试验结果，研究募集40例LS-SCLC和其他神经内分泌癌的患者，所有患者接受同步放化疗联合Pembrolizumab治疗，其中61%的患者接受预防性颅脑照射，结果显示中位无进展生存期（progression-free survival, PFS）达19.7个月，其95%可信区间（confidence interval, CI）为8.8个月-30.5个月，而中位OS为39.5个月（95%CI: 8.0-71.0）；患者总体毒副作用耐受良好。尽管单药免疫治疗的耐受性良好，但LS-SCLC患者的生存是否真正获益一直是焦点。2021年，Bilani等^[14]^对美国国家癌症数据库自2004年-2016年共50, 527例LS-SCLC患者进行分析，其中98例患者采用一线免疫治疗，79例可获得生存数据；与配对队列相比，免疫治疗并不能改善患者的长期生存，但该研究无法获取患者具体用药信息。STIMULI研究结果^[15]^显示，标准治疗后的LS-SCLC联合使用Nivolumab和Ipilimumab并没有达到改善PFS的终点，而且联合ICIs还带来一定的毒副作用升高。近期，一项针对Durvalumab在LS-SCLC中的单臂、Ⅱ期临床试验结果^[16]^公布，所有患者接受Durvalumab联合同步放化疗，后续维持Durvalumab治疗2年，其中位PFS为14.4个月（95%CI: 10.3-），中位OS尚未达到，2年PFS和OS率分别为42.0%、67.8%；总体疗效可观，耐受性高。ADRIATIC研究是一项拟评估Durvalumab单药或联合Tremelimumab对同步放化疗后LS-SCLC患者疗效的Ⅲ期、随机、双盲、安慰剂对照的全球多中心临床试验^[17]^，其结果尚未公布。

### 2.2 ES-SCLC的免疫治疗

相比于LS-SCLC，ES-SCLC更具有疗效提升的空间；目前SCLC的免疫治疗临床试验主要集中于ES-SCLC。目前无Tremelimumab单药免疫治疗的临床试验。

#### 2.2.1 Ipilimumab

Ipilimumab是最早应用于肺癌的ICI。一项评估Ipilimumab联合化疗对比单纯化疗在肺癌中有效性的Ⅱ期临床试验于2013年公布了初治ES-SCLC的研究结果^[18]^；患者随机接受化疗联合安慰剂或Ipilimumab治疗，结果表明，相比于单纯化疗，Ipilimumab阶段给药（而非同步给药）能够提高免疫治疗相关的生存期。然而，2016年公布的Ⅲ期临床试验（CA184-156）结果^[19]^却表明，Ipilimumab联合化疗对比安慰剂联合化疗作为ES-SCLC患者一线治疗方案并不能延长OS。因此，美国国家食品药品监督管理局（Food and Drug Administration, FDA）尚未批准Ipilimumab用于SCLC的治疗。

#### 2.2.2 Nivolumab

CheckMate 032研究是一项评估Nivolumab联合或不联合Ipilimumab在多种局部晚期或转移性实体肿瘤中安全性及有效性的Ⅰ期/Ⅱ期临床试验；Antonia等^[20]^于2016年公布了其在复发型SCLC的研究结果，研究设定了不同剂量的Nivolumab及Ipilimumab给药方案，结果表明，Nivolumab单药或者联合Ipilimumab具有抗肿瘤作用，而且毒副作用可控。短期研究结果使Nivolumab成为首个快速批准用于SCLC二线治疗的ICI。但CheckMate 331研究结果^[21]^却提示，对于复发型SCLC，二线使用Nivolumab并不能改善复发患者的OS；这导致了FDA撤除了Nivolumab针对既往治疗过的SCLC的适应证。对于维持免疫治疗，CheckMate 451研究结果^[22]^表明，ES-SCLC患者在接受一线以铂类为基础的化疗方案治疗有效后，序贯接受Nivolumab或Nivolumab联合Ipilimumab的维持治疗均不能延长OS；且双免疫联合模式的治疗相关副作用发生率更高；但亚组分析发现，肿瘤突变负荷≥13个突变/兆碱基的人群或许可以从双免疫维持治疗模式中获益。目前，Nivolumab已无FDA批准用于SCLC的适应证。

#### 2.2.3 Pembrolizumab

KeyNote028研究是一项评估Pembrolizumab在多种局部晚期或转移性实体肿瘤中安全性及有效性的Ⅰ期临床试验；2017年12月公布了24例ES-SCLC的研究结果^[23]^，8例患者出现3级-5级不良反应，其中2例与治疗相关；研究者评估的客观缓解率为33.3%；中位PFS为1.9个月（95%CI: 1.7-5.9），中位OS为9.7个月（95%CI: 4.1-）；短期内，Pembrolizumab在ES-SCLC中总体表现出一定的抗肿瘤活性，无新发毒性反应谱。2020年，Ⅲ期的Keynote604研究公布了Pembrolizumab联合化疗一线用于ES-SCLC的研究结果^[24]^，表明Pembrolizumab可使ES-SCLC患者获得更长的PFS，但其OS获益并不明显。基于此，FDA同样撤销了此前批准的Pembrolizumab在SCLC的适应证。2021年，一项针对既往治疗过的Ⅲ期/Ⅳ期SCLC的Ⅰ期多中心、非随机临床试验初步结果^[25]^表明Quavonlimab（CTLA-4抗体）联合Pembrolizumab具有一定的抗肿瘤作用，且毒性可耐受；期待进一步的临床试验结果。

#### 2.2.4 Atezolizumab

Impower133研究是一项评估化疗联合Atezolizumab或安慰剂在初治ES-SCLC中安全性和有效性的双盲、随机对照的Ⅲ期临床试验；2018年，新英格兰医学杂志发表的Impower133研究结果^[26]^，证实了相比于单用化疗，一线化疗联合Atezolizumab能够提高ES-SCLC患者的中位PFS（4.3个月-5.2个月）和中位OS（10.3个月-12.3个月）；2021年更新的生存数据^[27]^亦提示化疗联合Atezolizumab能够改善ES-SCLC的OS，而且毒副作用可耐受。此外Impower133研究的探索性分析结果^[28]^显示，化疗联合Atezolizumab后继续Atezolizumab单药维持治疗相比于仅仅化疗联合Atezolizumab，能进一步提高患者的PFS和OS。IFCT-1603研究是一项评估Atezolizumab对于一线化疗后进展的SCLC有效性的Ⅱ期临床试验，2019年其研究结果^[29]^表明，Atezolizumab单药用于复发的SCLC的二线治疗效果较化疗相比，并无生存获益。目前，FDA批准Atezolizumab与卡铂、依托泊苷联合用于成人ES-SCLC的一线治疗。

#### 2.2.5 Durvalumab

2019年柳叶刀杂志公布了针对ES-SCLC的Ⅱ期随机对照临床试验（CASPIAN研究）结果^[30]^，证实了一线化疗联合Durvalumab能使ES-SCLC患者获得OS的延长（中位OS：10.3个月-13.2个月）；研究方案包含了Durvalumab的维持治疗；2021年其公布的最新结果^[31]^进一步证实了Durvalumab联合化疗的一线治疗效果，但同时其结果表明Tremelimumab并不能改善此类患者预后。FDA批准Durvalumab联合依托泊苷和卡铂/顺铂用于成人ES-SCLC的一线治疗。

*Meta*分析结果^[32]^显示，SCLC的化疗联合免疫治疗总体反应率约33%-68%，疾病控制率约70%-88%；Pembrolizumab、Nivolumab、Atezolizumab和Durvalumab作为一线治疗方案，不同药物相互间的差异并不明显^[33]^。美国FDA批准ICIs用于SCLC的时间轴见图 1。SCLC免疫治疗临床试验的生存相关数据归纳于表 1。根据2022年第2版的美国国家癌症综合网络指南^[44]^，LS-SCLC的全身系统性治疗方案推荐4个疗程的依托泊苷和卡铂/顺铂方案，依托泊苷和卡铂方案被1类推荐用于化疗联合放疗。ES-SCLC的全身系统性治疗周期需要根据患者的反应及耐受性而定，一般为4个-6个疗程；其一线方案推荐4个疗程依托泊苷和卡铂方案联合Atezolizumab或Durvalumab后维持Atezolizumab或Durvalumab免疫治疗，或者依托泊苷和顺铂方案联合Durvalumab后维持Durvalumab免疫治疗。

**图 1:**
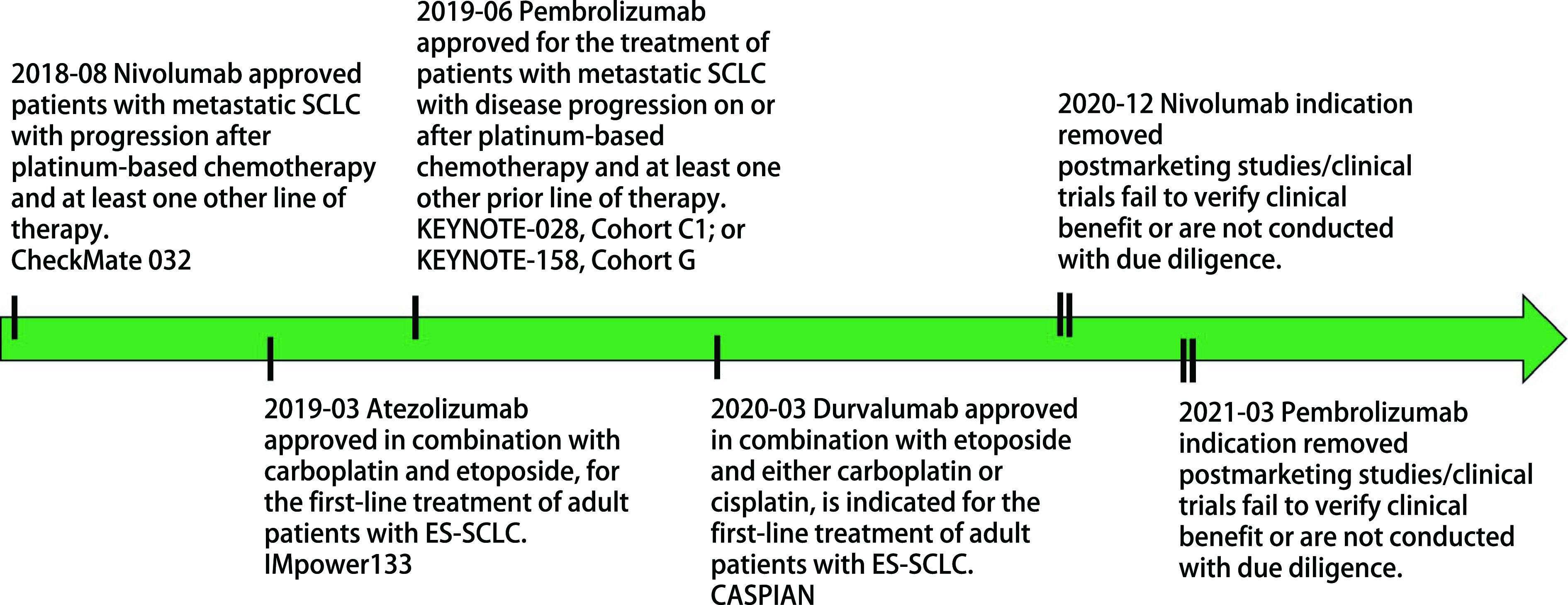
美国FDA批准免疫检查点抑制剂用于SCLC的时间轴 The timeline of FDA-approved immune checkpoint inhibitors for small cell lung cancer. SCLC: small cell lung cancer; ES-SCLC: extensive stage-SCLC; FDA: Food and Drug Administration.

**表 1:** SCLC的免疫治疗临床试验及结果 Clinical trials and results of immunotherapy for small cell lung cancer

Indication	Name/Number	Drug or treatment	Phase	Outcomes	Conclusion
LS-SCLC	STIMULI/ NCT02046733	N+I	2	median PFS: 10.7 mon (N+I) *vs* 14.5 mon (observation group)	N+I consolidation after CRT did not improve PFS in LS-SCLC^[15]^
	NCT03811002	P+CRT	1/2	median PFS: 19.7 mon; median OS: 39.5 mon	Concurrent CRT and P was well tolerated and yielded favorable outcomes^[13]^
	NCT03585998	D+CRT	2	median PFS: 14.4 mon; median OS: not reached; 24-mon OS: 67.8%	D with CRT for LS-SCLC exhibited promising clinical efficacy with a tolerable safety profile^[16]^
ES-SCLC	NCT 00527735	I+CT	2	CT+phased I *vs* CT+concurrent I *vs* CT median PFS: 5.2 mon, 3.9 mon, 5.2 mon median OS: 12.9 mon, 9.1 mon, 9.9 mon	Phased ipilimumab, but not concurrent ipilimumab, improved immune-related PFS *vs* control^[18]^
	NCT01450761	I+CT	3	I + CT *vs* CT median OS: 11.0 mon *vs* 10.9 mon; median PFS: 4.6 mon *vs* 4.4 mon	I+CT did not prolong OS in newly diagnosed ES-SCLC. No new adverse events were observed^[19]^
	CheckMate 032/ NCT01928394	N or N+I	1/2	2016 (N *vs* N + I) ORR: 13% *vs* 31%; 1-year OS: 27% *vs* 48% median OS: 3.55 mon *vs* 7.75 mon; median PFS 1.38 mon *vs* 3.35 mon 2020 (N *vs* N + I) ORR: 11.6% *vs* 21.9%; 24-mon OS: 17.9% *vs* 16.9% median OS: 5.7 mon *vs* 4.7 mon	2016: N or N+I showed antitumor activity and manageable safety profiles in previously treated SCLC^[20]^ N and N+I showed durable ORR with possibly higher toxicities observed with the combination^[34]^ 2018: N+I provide a greater clinical benefit than N in the high tumor mutational burden tertile^[35]^ 2019: N monotherapy provided durable responses as a third- or later-line treatment for recurrent SCLC^[36]^ 2020: OS was similar between groups. Toxicities were more common with combination therapy^[37]^
	CheckMate 451/ NCT02538666	N or N+I	3	median OS N+I: 9.2 mon; N: 10.4 mon; placebo: 9.6 mon	Maintenance therapy with N+I did not prolong OS in ES-SCLC^[22]^
	NCT03662074	Gemcitabine+N	2	PD: 78.6%, PR: 7.1%; median PFS 1.8 mon	No article published
	KEYNOTE028/ NCT02054806	P	1	ORR: 33% with P monotherapy median PFS: 1.9 mon; median OS: 9.7 mon	P demonstrated promising antitumor activity with the known safety profile in pretreated PD-L1-expressing SCLC^[23]^
	KEYNOTE-604/ NCT03066778	P+CT	3	12-mon PFS estimates: 13.6% (P+CT) *vs* 3.1% (CT) 24-mon OS estimates: 22.5% (P+CT) *vs* 11.2% (CT) ORR: 70.6% (P+CT) *vs* 61.8% (CT)	First-line P+CT significantly improved PFS for patients with ES-SCLC. No unexpected toxicities^[24]^
	REACTIon/ NCT02580994	P+CT	2	P+CT *vs* CT median PFS: 4.7 mon *vs* 5.4 mon; median OS: 12.3 mon *vs* 10.4 mon response rate: 61% (67% *vs* 56%)	P+CT was well tolerated but did not improve PFS over CT in chemo-sensitive patients with untreated ES-SCLC^[38]^
	IMpower133/NCT02763579	EC±A	3	EC+A *vs* EC 2018 median OS: 12.3 mon *vs* 10.3 mon; median PFS: 5.2 mon *vs* 4.3 mon 2021 median OS: 12.3 mon *vs* 10.3 mon; 18-mon OS: 34.0% *vs* 21.0%	2018: A+CT in the first-line treatment of ES-SCLC resulted in longer OS and PFS than CT^[26]^ 2020: A+EC had a comparable safety profile to placebo+EC^[40]^ 2021: First-line A+EC for ES-SCLC continued to improve OS with tolerable safety profile^[27]^
	NCT03059667	A *vs* CT	2	A *vs* CT median PFS: 1.4 mon *vs* 4.3 mon; median OS: 9.5 mon *vs* 8.7 mon	A monotherapy in relapsed SCLC failed to show significant efficacy^[29]^
	CASPIAN/NCT03043872	D±T±CT	3	median OS 2019 D+CT: 13.0 mon; CT: 10.3 mon 2021 CT: 10.5 mon; D+CT: 12.9 mon; D+T+CT: 10.4 mon	2019 First-line D+CT significantly improved OS in patients with ES-SCLC *vs* control group^[30]^ 2021 First-line D+CT sustained OS improvement but the addition of T didn’t improve outcomes^[31]^
Relapsed or refractory SCLC	CheckMate 331/ NCT02481830	N *vs* CT	3	N *vs* CT median OS: 7.5 mon *vs* 8.4 mon; median PFS: 1.4 mon *vs* 3.8 mon ORR: 13.7% *vs* 16.5%	N did not improve survival *vs* CT in relapsed SCLC. No new safety signals were seen^[21]^
	NCT02551432	P+paclitaxel	2	ORR: 23.1%; DCR: 80.7% median PFS: 5.0 mon; median OS: 9.1 mon	P and paclitaxel combination in refractory SCLC showed moderate activity with acceptable toxicity^[39]^
ES-SCLC (combination with radiotherapy)	NCT03043599	I+N+Thoracic RT	1/2	6-mon PFS: 24%; 12-mon OS: 48% median PFS: 4.5 mon; median OS: 11.7 mon	I+N after CT and TRT did not significantly improve PFS, the OS was higher than expectations^[41]^
	NCT02402920	P+CRT	1	median PFS: 6.1 mon; median OS: 8.4 mon	Concurrent pembrolizumab-RT was tolerated well^[42]^
	NCT02701400	T+D±RT	2	T+D *vs* T+D+SBRT median PFS: 2.1 mon *vs* 3.3 mon; median OS: 2.8 mon *vs* 5.7 mon ORR: 0% *vs* 28.6%	T and D combination±SBRT was safe but did not show sufficient efficacy in relapsed SCLC^[43]^
Relapsed ES-SCLC (new drugs)	NCT03026166	Rovalpituzumab Tesirine and N±I	1/2	ORR: 27.6% *vs* 36.4% PFS: 4.8 mon *vs* 4.1 mon; OS: 7.4 mon *vs* 11.0 mon	Terminated (Enrollment was stopped after the DLT in evaluation phase)
	NCT03179436	Quavonlimab+P	1	ORR: 18%; median PFS: 2.0 mon; median OS: 11.0 mon	Encouraging antitumor activity was observed with quavonlimab+P in ES-SCLC^[25]^
A: Atezolizumab; D: Durvalumab; I: Ipilimumab; N: Nivolumab; P: Pembrolizumab; T: Tremelimumab; CT: chemotherapy; CRT: chemoradiotherapy; DLT: dose-limiting toxicity; EC: carboplatin-etoposide; ORR: objective response rate; OS: overall survival; RT: radiotherapy; SBRT: stereotactic body radiation therapy; SOC: standard of care; DCR: disease control rate; PFS: progression-free survival; *vs*: versus.					

### 2.3 SCLC免疫治疗研究前景

目前，大量的注册临床试验正积极开展，以进一步评估ICIs在SCLC中的有效性及安全性；其中针对LS-SCLC的临床试验主要涉及联合化疗的初始免疫治疗及标准治疗后的免疫维持治疗等，针对ES-SCLC的临床试验主要包含ICIs新药以及不同的联合治疗模式等。尽管国产ICIs目前尚无SCLC的临床适应证，可喜地是，Sintilimab（NCT04189094）、Toripalimab（NCT04418648）、SHR-1316（NCT04647357）以及SHR1210（NCT04790539）等正广泛开展相关临床试验。

除上述常见免疫检查点外，新型免疫检查点如TIGIT、VISTA、LAG3等不断被发现并逐渐开发出ICIs药物，如TIGIT抗体Ociperlimab和Tiragolumab等已在SCLC人群中开展联合PD-1/PD-L1的免疫治疗研究（NCT04952597, NCT04256421）。此外，ICIs联合抗血管生成药物、新型靶向药物以及癌症疫苗的临床研究亦不断涌现。SCLC作为神经内分泌癌的一种，借鉴于其他神经内分泌癌的治疗经验，目前多项研究正考虑ICIs联合Temozolomide（NCT03728361）、177Lu-DOTA0-Tyr3-Octreotate（NCT03325816）等针对神经内分泌肿瘤的药物。针对含ICIs的联合治疗，我们一方面需关注抗肿瘤的临床疗效，另一方面仍需警惕免疫治疗相关的不良反应。CheckMate 032研究^[37]^提出联合免疫治疗的副作用较单药更常见。一项评估Rovalpituzumab Tesirine（DLL3抗体偶联药物）和Nivolumab联合或不联合Ipilimumab在ES-SCLC人群中安全性的临床试验（NCT03026166）因剂量限制毒性而导致试验提前终止。药物靶点与药物剂量可能是免疫相关不良反应的关键。

此外，放疗联合免疫治疗也广为关注。放疗联合免疫治疗的协同作用在动物试验中被发现^[45, 46]^，放疗或许能够增强ICIs的抗肿瘤作用^[47]^。Pembrolizumab同步联合胸部放疗用于诱导化疗后的ES-SCLC研究^[42]^中，无治疗相关的4级-5级毒性反应发生，整体毒副作用可控；尽管与既往报道相比，可能存在一定的生存获益，但由于随访时间较短且为单臂Ⅰ期研究，故而在生存分析上存在一定的缺陷。Ipilimumab、Nivolumab联合胸部放疗应用于含铂类双药化疗的ES-SCLC的研究^[41]^未报道新发的毒性反应谱，但并未延长患者的PFS。一项评估Durvalumab和Tremelimumab联合或不联合立体定向放疗用于复发型SCLC的随机、双臂的Ⅱ期临床试验^[43]^虽然提示Durvalumab和Tremelimumab联合立体定向放疗具有生存获益的趋势，但分析结果无统计学差异。ICIs联合放疗或许需要寻找更合适的人群。

## 3 SCLC基因分型与免疫治疗

绝大多数SCLC均存在抑癌基因*TP53*及*RB1*的失活，但肿瘤的异质性却始终存在。起初，SCLC被分为经典型和变异型^[48]^；后来，根据肿瘤细胞是否具有神经内分泌分化特征将SCLC分为神经内分泌型和非神经内分泌型。近些年，针对SCLC肿瘤组织、细胞系以及基因工程小鼠模型的研究逐渐发现SCLC存在四种关键基因的突变，即*ASCL1*、*NEUROD1*、*YAP1*和*POU2F3*；Rudin等^[49]^于2019年提出，根据此四种不同的基因突变类型，将SCLC分为四个分子亚型，即SCLC-A、SCLC-N、SCLC-Y和SCLC-P，其中SCLC-A最为常见。

Gay等^[50]^发现，YAP1蛋白的表达在其他亚型SCLC中存在重叠；而这类肿瘤虽然缺乏*ASCL1*、*NEUROD1*和*POU2F3*基因突变，但表达多种免疫检查点以及人类白细胞抗原，免疫检查点包括CD274（PD-L1）、PDCD1（PD-1）以及CTLA-4及其受体；因此将这类疾病归为SCLC-炎症型或SCLC-I；SCLC-I型肿瘤中高表达CD8A和CD8B，肿瘤周围存在较多的免疫细胞浸润；Gay等^[50]^对IMpower133研究的人群进行亚型分析时发现，SCLC-I型人群在Atezolizumab联合化疗组中具有更高的OS，提示SCLC-I亚型或许更能从ICIs的治疗中获益。

## 4 SCLC疫苗相关免疫治疗

除了早期的卡介苗免疫治疗临床试验^[12]^，Giaccone等^[51]^于2005年报道了卡介苗与Bec2联合用于LS-SCLC的Ⅲ期临床试验，卡介苗与Bec2混合使用能够刺激免疫系统产生更多的GD3抗体，从而起到抑制肿瘤细胞增殖的作用；但研究最终结果却显示两组患者的OS、PFS并无显著差异。多唾液酸（polysialic acid, polySA）常在SCLC中表达，而除了脑组织，其他正常组织中基本不表达。Krug等^[52, 53]^系列研究表明，polySA疫苗在SCLC细胞系或人体试验中可引起相应的免疫反应，但其临床上的生存结局尚不确定。个体化肽类疫苗是癌症疫苗免疫治疗的另一个方向；根据人类白细胞抗原研发设计的肽类疫苗，能够引起T细胞的富集，增强对肿瘤细胞的杀伤作用。个体化肽类疫苗的Ⅱ期临床试验^[54]^纳入46例SCLC患者，疫苗注射后能够引起显著的免疫球蛋白G的反应，同时出现了临床患者OS的延长，但该研究未对SCLC的分期进行分层分析。*TP53*作为SCLC常见的抑癌基因突变，已有多项关于TP53的疫苗研究。Antonia等^[55]^在2006年报道了使用腺病毒载体转染全长野生型*p53*基因组成的癌症疫苗应用于ES-SCLC的研究，57.1%的患者产生了p53特异性的T细胞疫苗反应，而且二线化疗联合疫苗人群的客观临床缓解率达61.9%，提示化疗联合癌症疫苗具有一定的应用前景；其团队于2013年报道了另外一项关于癌症疫苗的临床研究（NCT00617409）结果^[56]^，研究将标准一线化疗后疾病稳定的ES-SCLC患者随机入组，分为标准治疗对照/观察组（18例）、p53疫苗单药组（19例）以及p53疫苗联合全反式维甲酸治疗组（19例）；疫苗采用表达p53的腺病毒转载的树突状细胞；结果显示，p53疫苗联合全反式维甲酸治疗组能够降低2倍以上的髓源性抑制细胞浓度，但p53疫苗单药组并无明显效应发生；类似的结果亦可见于p53特异性的免疫反应；遗憾的是，该团队于2019年公布的临床结局^[57]^显示，作为二线治疗，尽管药物总体安全性尚可，但三组间并无显著的生存差异。2021年，Gonzalez-Cao等^[58]^报道了Atezolizumab联合树突状细胞疫苗的临床研究方案，目前正处于患者入组阶段。Durvalumab正开展一项联合个体化新抗原DNA疫苗用于ES-SCLC的临床研究（NCT04397003），我们期待其相关结果。针对其他分子靶点的新抗原疫苗的研究可见于NSCLC的研究，但尚未见SCLC的报道。

## 5 小结

SCLC侵袭性强，预后差。明确病理类型、判定肿瘤分期，对于SCLC而言至关重要。化疗联合免疫治疗是ES-SCLC的首选治疗方案。对于复发进展疾病、标准治疗后的维持治疗方案，仍需更多的临床试验证据说明免疫治疗的获益与否及耐受性。我们期待新型免疫治疗药物以及包含免疫治疗的多药物联合应用的临床研究结果。
